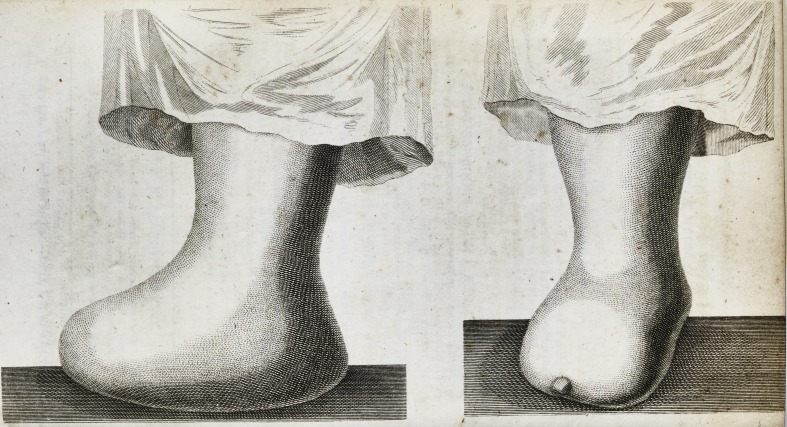# Case of Amputation at the Instep

**Published:** 1817-05

**Authors:** John C. Yeatman

**Affiliations:** Member of the Royal College of Surgeons, &c.


THE LONDON

### Medical and Physical Journal.

5 OF VOL. XXXVII.] MAY, 1817. [NO. 219.

"For many fortunate discoveries in medicine, and for the detection of numerous errors, the world is indebted to the rapid circulation of Monthly Journals; and there never existed any work to which the Faculty in EUROPE and AMERICA were under deeper obligations than to the Medical and Physical Journal of London, now forming a long, but an invaluable, series."—RUSH.

*For the London Medical and Physical Journal.*

### *Case of Amputation at the Instep, continued from Page 359, (with an Engraving.)* By JOHN C. YEATMAN, Esq. Member of the Royal College of Surgeons, &c.

MOST hospital-surgeons have removed portions, and even one or two entire bones of the metatarsus, under circumstances of injury or of disease; but Chopart and Villeorne, and Hey of Leeds, are, I believe, the only surgeons who have recorded cases in which complete amputation at the instep has been performed; and, as those gentlemen have been successful in their operations, it is to be hoped they will be followed by others.

I am indebted to Amicus, in your last Number, for informing me, that Mr. Hey had amputated at the instep: it may appear strange, that I was not aware of the fact; but, having been from England for some years lately, I had no means of seeing Mr. H.'s excellent Practical Observations in Surgery. On referring to that work, it would seem, that the author was totally unacquainted with M. Chopart's operation, first published in 1792, five years before Mr. H. amputated at the instep; so that the latter gentleman may be considered also original in this improvement.

Having already described the operation in the case of Sarah Slade, and the quarter-boot which she has worn with so much advantage, I have now only to add the few following remarks.

The length of the foot from the heel to the root of the little toe was three inches and three-quarters,—only three-fourths of an inch more than from the heel to the stump.

The mark on the stump is intended to show the small cicatrix over the os cuboides, or, to be exact, I would rather say, over the junction of the cuboid and external cuneiform bone, at which joint the ligature passed out.

 The

The fulness visible in the sole of the foot was prior to the operation, and occasioned by the throwing out of coagulated lymph during a more than sufficient effort in the reproduction of parts subsequent to sloughing.

It may be observed, that, although the line of separation between the dead and living parts prevented my leaving as much flap in the operation as in Mary Stansfield's case, (of which Mr. Hey has given an engraving,) yet enough was preserved to form a good cushion over the whole anterior surface of the tarsus; and it will, of course, be understood, that those granulations which were generated around the edges of the sore after sloughing were pared away, with the view to adhesion in the stump.

In performing this operation, I very much question whether it would not be better to retain the base and tuberosity of the metatarsal bone of the little toe, by applying the saw transversely two or three lines anterior to its articulation with the cuboid, as, besides preserving the lesser arch of the foot, and increasing the base of support to the inferior limb, the peroneus brevis and peroneus tertius of Albinus would not be set at liberty,—an objection which does not apply to either of the other ossa metatarsa; for, with the exception of the adductor pollicis, those muscles that are inserted into their posterior extremities are likewise attached to the tarsal bones. The operator also would be spared the trouble of dissecting it from its strong attachments and angular conjunction with the os cuboides.

*Frome.*

---

*For the London Medical and Physical Journal.*

## Figures and Tables

**Figure f1:**