# Errata

**Published:** 1817-06

**Authors:** 


					Vol. 37, page 353, line 3, for Villeorne read Vilierme.?Same page, last
line, for joint read point.-?Pane 354, line 2, for coagulated read coagulable.

				

